# Spermidine Inhibits M1 Microglia Polarization in a Mouse Model of Parkinson's Disease and BV2 Cells via NF‐κB/STAT‐1 Pathway

**DOI:** 10.1002/brb3.70410

**Published:** 2025-03-09

**Authors:** Jun Shu, Yuqiong Jiao, Wenshi Wei, Aijuan Yan

**Affiliations:** ^1^ Department of Neurology Huadong Hospital, Fudan University Shanghai China

**Keywords:** microglial polarization, neuroinflammation, Parkinson's disease, spermidine

## Abstract

**Background:**

Excessively activated M1 microglia release proinflammatory factors that can cause neuronal death and contribute to the development of Parkinson's disease (PD). Recent research indicates that spermidine, a naturally occurring polyamine, may have anti‐inflammatory properties. Nonetheless, the specific role of spermidine in Parkinson's disease, particularly how it affects microglia‐driven neuroinflammation and the balance between M1 and M2 polarization, is still not fully understood.

**Methods:**

We examined the effects of spermidine on the polarization of M1/M2 microglia in a 1‐methyl‐4‐phenyl‐1,2,3,6‐tetrahydropyridine (MPTP) mouse model of PD and lipopolysaccharide (LPS)‐stimulated BV2 cells. Methods like RT‐PCR, western blotting, and immunofluorescence were used to examine how spermidine influences the polarization of microglia.

**Results:**

In vivo, spermidine pretreatment reduced the activation of M1 microglia and encouraged the transformation of microglia into the M2 phenotype in the substantia nigra (SN) of PD mice. Additionally, spermidine decreased the release of inflammatory factors and lessened the death of dopaminergic neurons in the SN of these mice. In vitro, spermidine indirectly protected neurons from death by affecting microglial polarization. Furthermore, spermidine preconditioning led to decreased phosphorylation of NF‐κB, STAT1, and p38 MAPK, while enhancing the phosphorylation of STAT6, both in vivo and in vitro. Additionally, we observed that the supernatant from BV2 cells was cultured with SH‐SY5Y neurons. The findings revealed that the supernatant from LPS‐activated BV2 cells notably reduced the viability of SH‐SY5Y cells, as well as the levels of brain‐derived neurotrophic factor (BDNF), TrkB, PI3K, and p‐AKT. However, these effects were significantly reversed by pretreatment with spermidine.

**Conclusion:**

Our research found that spermidine reduced M1 microglial polarization, partially through the inhibition of the NF‐κB, STAT1, and p38 MAPK pathways, and encouraged M2 microglial polarization by activating the STAT6 pathway. This action helped to mitigate neuroinflammation in both the MPTP mouse model of Parkinson's disease and LPS‐stimulated BV2 cells. Additionally, spermidine provided indirect neuroprotection by activating BDNF‐TrkB‐PI3K/AKT signaling pathways.

## Graphical abstract

Spermidine pretreatment reduced M1 microglial polarization and promoted M2 microglial polarization by inhibiting the NF‐κB/STAT1 signaling pathways, thereby alleviating neuroinflammation in both the MPTP mouse model of Parkinson's disease and LPS‐stimulated BV2 cells. Furthermore, spermidine indirectly inhibited neuronal apoptosis by activating the BDNF‐TrkB‐PI3K/AKT signaling pathways, offering neuroprotection.

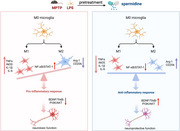

## 1 Introduction

Parkinson's disease (PD) is a multisystem disorder impacting various brain regions and leading to significant loss of dopaminergic, noradrenergic, and serotonergic neurons (Lobb 2014). The etiology of PD remains unclear, but mitochondrial dysfunction, oxidative stress, and neuroinflammation are involved (Lee et al. 2017), and neuroinflammatory responses related to microglial activation are implicated during the death of dopaminergic neurons (Yan et al. 2018).

Microglia are key components of the mononuclear phagocyte system in the central nervous system (CNS) and exhibit diverse functions throughout different life stages, influencing both physiological processes and pathological response (Boche et al. 2013). When microglia become overactivated, they release inflammatory cytokines, resulting in chronic neuroinflammation. This neuroinflammatory response is associated with the progression of PD (Rocha et al. 2015). Research indicates that microglial activation in the CNS can be categorized into M1 and M2 phenotypes. Depending on their activation state, microglia can either release cytotoxic factors or provide neuroprotection (Tang and Le 2016). M1 microglia, which are classically activated, produce elevated levels of pro‐inflammatory cytokines such as interleukin‐6 (IL‐6), IL‐1β, interferon gamma (IFN‐γ), and tumor necrosis factor alpha (TNF‐α) (Boche et al. 2013; Butturini et al. 2019). Neuroinflammatory response can be induced by M1 microglia through nuclear factor‐ κB (NF‐κB) p65, STAT1, and p38 mitogen‐activated protein kinase (MAPK) signaling pathways (Du et al. 2018; B. Liu et al. 2021). M2 microglia play a crucial role in tissue repair and the suppression of neuroinflammation, and their activation is induced by IL‐4. IL‐4 binds to specific receptor complexes, triggering the activation of JAK1 or JAK3, which in turn activates STAT6 and promotes the transcription of M2‐related genes, such as CD206 (Subramaniam and Federoff 2017). Considering the functional plasticity of microglial cells, recent studies on CNS injury treatment have emphasized encouraging a transition from the M1 to the M2 phenotype, with promising outcomes (B. Zhang et al. 2015)

Spermidine is a naturally occurring polyamine derived from arginine through the action of arginase, and it is found in high concentrations in the brain (Velloso et al. 2008). Reduced levels of polyamines, including spermidine, have been associated with aging and neurodegenerative disorders (Y. Zhang et al. 2017). Numerous studies have demonstrated spermidine's anti‐aging, antioxidant, and anti‐inflammatory properties, suggesting it could be beneficial in treating such conditions (Ghosh et al. 2020; Jeong et al. 2018; Minois 2014). For instance, research has shown that spermidine can enhance the anti‐inflammatory response of peripheral macrophages, effectively reducing autoimmune encephalomyelitis (EAE) (Yang et al. 2016). In line with earlier research, spermidine has been shown to diminish inflammation in macrophages stimulated by lipopolysaccharide (LPS) in vitro (Jeong et al. 2018; R. Liu, Li, et al. 2020). Additionally, spermidine appears to inhibit neuroinflammation in microglia by disrupting the inflammasome (Freitag et al. 2022). However, the specific molecular mechanisms or signaling pathways through which spermidine mediates its anti‐inflammatory effects in the central nervous system remain unclear.

The decline in tissue spermidine levels with age, observed in both model organisms and humans (Madeo et al. 2018), may contribute to increased neuroinflammation in PD. The potential of spermidine pretreatment to mitigate neuroinflammation in a 1‐methyl‐4‐phenyl‐1,2,3,6‐tetrahydropyridine (MPTP)‐induced PD model has not yet been explored. Based on existing evidence, the hypothesis is that spermidine pretreatment could reduce neuroinflammatory processes and microglial activation in PD. To test this, our study aims to investigate spermidine's effects on neuroinflammation and microglia modulation in both an MPTP‐induced PD model and an LPS‐stimulated BV2 microglial cell line.

## 2 Material and Methods

### 2.1 Reagents and Mice

LPS, spermidine, and MPTP were procured from Sigma‐Aldrich Co. in St. Louis, MO, USA, whereas IL‐4 was acquired from PeproTech, USA. Male C57BL/6 mice, 10‒12 weeks old, were obtained from Shanghai SLAG Laboratory Animal Corporation. The mice were housed under standardized conditions featuring a 12‐h light/dark cycle, a room temperature of 22 ± 1°C, and 50%‒60% relative humidity. They had continuous access to both food and water. The study was approved by the Ethics Committee of Huadong Hospital, Fudan University, and followed the ARRIVE guidelines for in vivo research.

### 2.2 Cell Culture and Treatment

The BV2 microglial and SH‐SY5Y neuronal cell lines were obtained from the China Center for Type Culture Collection. Both cell types were cultured in DMEM supplemented with 10% fetal bovine serum (FBS) and 1% penicillin/streptomycin, and incubated at 37°C in a humidified environment with 95% air and 5% CO₂. Spermidine was initially dissolved in PBS to create a 1 M stock solution and then diluted in DMEM for application. In the experiments, BV2 cells were pretreated with different concentrations of spermidine for 1 h before being exposed to LPS (100 ng/mL) for 2, 12, or 24 h.

### 2.3 TUNEL Staining and CCK‐8 Assay

To investigate the impact of conditioned media from spermidine‐treated BV2 cells on SH‐SY5Y neuronal cell death, we conducted TUNEL and CCK‐8 assays. Initially, BV2 cells were cultured in 6‐well plates and exposed to 0.8 µM spermidine for 1 h, followed by a 24‐h incubation with LPS. The supernatants from these BV2 cells were then collected and used to treat SH‐SY5Y cells for 24 h. After treatment, SH‐SY5Y cells were fixed with 4% paraformaldehyde (PFA). Following fixation, cells were washed with PBS and subjected to the TUNEL assay as per the provided protocol. The cell viability was evaluated using the CCK‐8 assay, conducted following the manufacturer's instructions. This assay measures cell metabolic activity as an indicator of cell viability and proliferation.

### 2.4 Induction and Treatment of PD Model

To create the MPTP‐induced Parkinson's disease (PD) model, the protocol followed was similar to that described in our previous research (Yan et al. 2018). The mice were administered 80 mg/kg MPTP at a rate of 20 mg/kg every 2 h for 8 h. To evaluate the protective effect of spermidine on PD, spermidine (at doses of 5 and 10 mg/kg) or saline was administered intraperitoneally daily, starting on Day 7 before the MPTP injections. The animals were euthanized on Days 1, 3, 5, 7, and 14 following the final MPTP injection. Brain tissues were collected for western blot, immunohistochemistry and RT‐PCR analysis to investigate various molecular and cellular aspects.

### 2.5 Open Field Test

The open‐field test involves placing the animals in a large, open arena and measuring various parameters such as distance traveled, movement speed, and time spent in different areas of the arena. This helps in evaluating changes in locomotor activity and overall motor function. On Days 3 and 7 after MPTP injection, motor behavior was recorded for 5 min and analyzed using SuperMaze V2.0 software. This software helps quantify various aspects of locomotion and motor activity by analyzing the recorded behavior data, providing insights into the effects of the MPTP treatment on motor function over time.

### 2.6 Pole Test

The pole test was used to assess motor function as outlined in our prior research (Yan et al. 2018). Mice were trained for the task three days prior to testing. Each mouse was placed at the top of a 55 cm long, 10 mm diameter pole and tested three times. The average time taken for the mice to turn and descend was recorded for statistical analysis.

### 2.7 Staircase Test

Staircase test is a behavioral test employed to assess motor function, particularly in terms of dexterity and coordination (Ugwah‐Oguejiofor et al. 2024). The experimental apparatus consisted of an acrylic box, measuring 45 cm in length, 10 cm in width, with one end raised to 12.5 cm and the other end to 25 cm. Inside the box, there were five identical stair steps, each 2.5 cm in height and 10 cm in width. During the experiment, the mouse was placed at the bottom of the box, facing the stairs. The numbers of steps climbed (defined as using all four limbs to ascend) and rearing were counted for 3 min. Rearing was recorded when the mouse rose on its hind legs either on the step or against the wall to sniff the air. The experiment was conducted in a quiet environment with constant lighting.

### 2.8 Immunohistochemistry and Double Immunofluorescence

The animals underwent transcardial perfusion with a solution of 4% paraformaldehyde (PFA) in 0.1 M phosphate‐buffered saline (PBS). Frozen sections of mouse brain tissue were obtained. The brain tissue sections were incubated in the following primary antibodies: rabbit anti‐TH (1:500, Abcam); rabbit anti‐Iba‐1 (1:200, Abcam); goat anti‐CD206 (1:100, R&D systems); mouse anti‐CD16/32 (1:200, BD Pharmingen) in Immunofluorescence Staining Antibody Dilution Buffer (Solarbio) for 24 h at 4°C. Following washing, the sections were treated with secondary antibodies—donkey anti‐goat, donkey anti‐rabbit, or donkey anti‐mouse—conjugated to Alexa Fluor‐594 or Alexa Fluor‐488 fluorochromes (1:200 dilution, Life Technologies) for 1 h at room temperature. Immunostaining of BV2 cells was carried out following a similar procedure. Both brain sections and BV2 cells were examined with an LSCM confocal microscope to obtain relevant images. For quantitative analysis, the number of immunopositive cells was counted in every fifth section (20 µm thick) of the substantia nigra (SN) or in five representative areas of the cell cultures.

### 2.9 RNA Isolation and Quantitative RT‐PCR

Total RNA was isolated with TRIzol reagent (Takara, Japan) following the manufacturer's guidelines. For cDNA synthesis, the RNA was reverse‐transcribed using the PrimeScript RT Reagent kit (Takara, Japan) as described in our earlier method (Yan et al. 2017). RT‐PCR was conducted using the Bio‐Rad CFX96 Detection System with SYBR Green qPCR Master Mix (Thermo Fisher Scientific). Expression levels of the target genes were normalized to ribosomal phosphoprotein P0 (Rplp0). The primers used for RT‐PCR are showed in Table 1.

**TABLE 1:** Primers for real‐time polymerase chain reaction.

name	For (5′–3′)	Rev (5′–3′)
iNOS	ATGTCCGAAGCAAACATCAC	TAATGTCCAGGAAGTAGGTG
IL‐1β	GCAACTGTTCCTGAACTCAACT	ATCTTTTGGGGTCCGTCAACT
IL‐6	TAGTCCTTCCTACCCCAATTTCC	TAGTCCTTCCTACCCCAATTTCC
TNF‐α	CCCTCACACTCAGATCATCTTCT	GCTACGACGTGGGCTACAG
CD16	TTTGGACACCCAGATGTTTCAG	GTCTTCCTTGAGCACCTGGATC
CD32	AATCCTGCCGTTCCTACTGATC	GTGTCACCGTGTCTTCCTT GAG
CD86	TTGTGTGTGTTCTGG AAACGGAG	AACTTAGAGGCTGTG TTGCTGGG
CD11b	CCAAGACGATCTCAGCATCA	TTCTGGCTTGCTGAATCCTT
Arg‐1	GAACACGGCAGTGGCTTTAAC	TGCTTAGCTCTGTCTGCTTTGC
CD206	TCTTTGCCTTTCCCAGTCTCC	TGAC ACCCAGCGGAATTTC
YM‐1	CAGGGTAATGAGTGGGTTGG	CACGG CACCTCCTAAATTGT
IL‐10	GCTCCAAGACCAAGGTGTCTACAA	CCGTTAGCTAAGATCCCTGGATCA
TGF‐β	TGCGCTTGCAGAGATTAAAA	CGTCAAAAGACAGCCACTCA
Rplp0	AGATTCGGGATATGCTGTTGGC	TCGG GTCCTAGACCAGTGTTC

### 2.10 Western Blot Analysis

Proteins from cell and brain samples (30 µg each) were resolved on 10%–12.5% SDS‐PAGE gels and subsequently transferred to PVDF membranes at 300 mA for 40–80 min. The membranes were then incubated with 5% non‐fat milk in TBST for 1 h to block non‐specific binding. Following this, they were incubated overnight at 4°C with primary antibodies against NF‐κB P65 (1:1000, #8242, CST), p‐NF‐κB P65 (1:1000, #3033, CST), STAT1 (1:1000, #9172, CST), p‐STAT1(1:1000, #9167, CST), STAT6 (1:1000, #9362, CST), p‐STAT6 (1:1000, #9361, CST), p38 MAPK (1:1000, #9212, CST), p‐p38 MAPK (1:1000, #9211, CST), TrkB (1:1000, #4603, CST), P‐AKT(1:1000, #9271, CST), AKT(1:1000, #9272, CST), BDNF (1:1000, #ab108319, Abcam), PI3K (1:200, sc‐365404, Santa Cruz), TH (1:500, #9167, Abcam), and β‐actin (1:1000, sc‐81178, Santa Cruz, USA). Following three washes with TBST, the membranes were exposed to anti‐rabbit or anti‐mouse secondary antibodies for 1 h at room temperature. Protein bands were detected using an enhanced chemiluminescence (ECL) detection kit (Millipore).

### 2.11 Statistical Analysis

All cell experiments were carried out in triplicate, and each animal group included a minimum of three mice. Data analysis was performed with GraphPad Prism software, using one‐way ANOVA and Tukey's multiple comparison test to assess significance. Results with *p* values less than 0.05 were deemed statistically significant. Data are shown as mean ± standard error of the mean (SEM).

## 3 Results

### 3.1 Dynamic Changes in M1 and M2 Microglial Phenotypes in the SN of PD Mice

**FIGURE 1:**
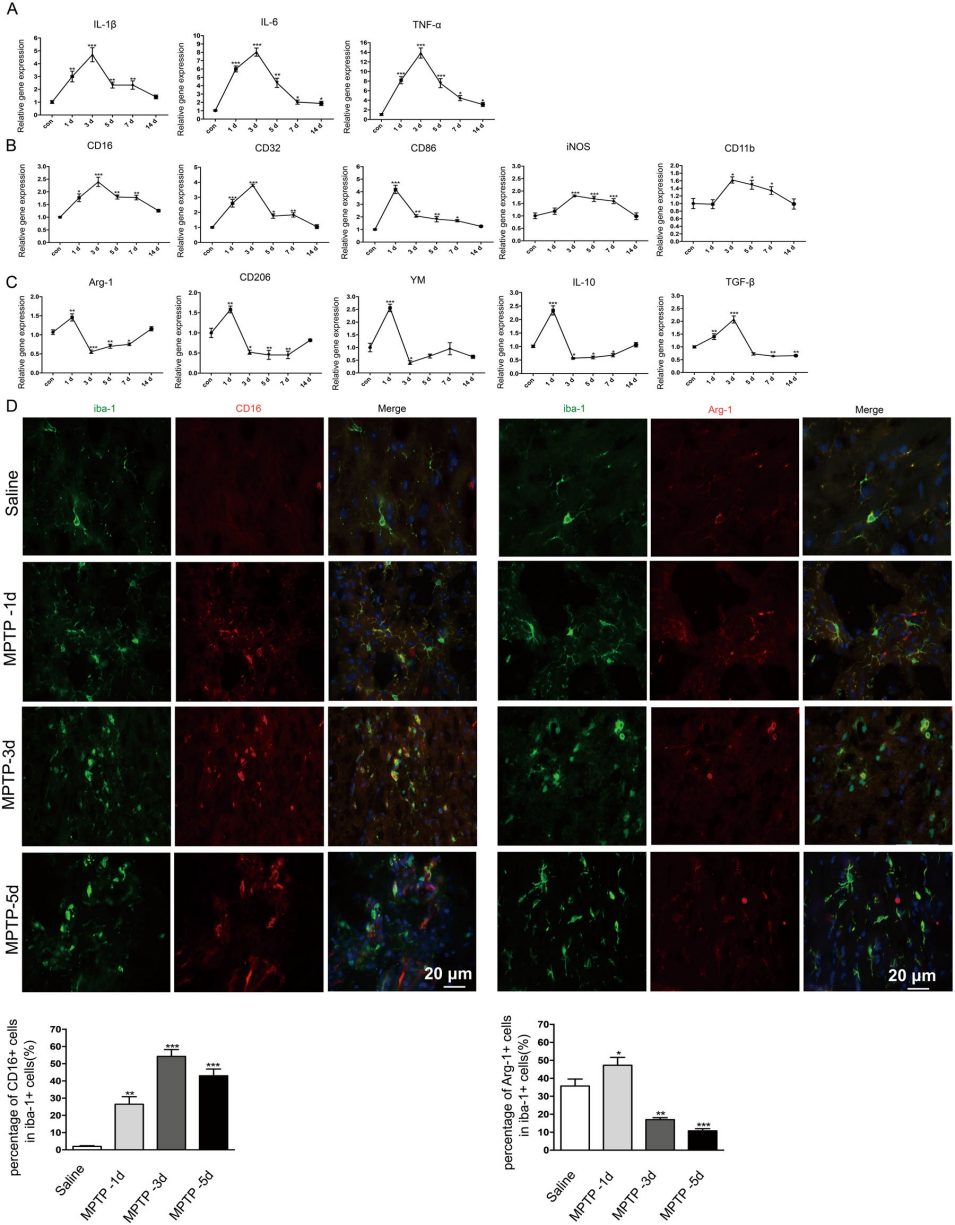
Temporal changes in M1 and M2 phenotype microglia in the substantia nigra (SN) of PD mice following MPTP administration. (A) Expression of proinflammatory factors (IL‐1β, IL‐6, and TNF‐α) in SN; (B) Expression of M1 markers (CD16, CD32, CD86, iNOS, and CD11b) in SN; (C) Expression of M2 microglial markers (Arg‐1, CD206, YM‐1, IL‐10, and TGF‐β) in SN of PD mice as determined by RT‐PCR on Days 1, 3, 5, 7, and 14 after MPTP administration. *p < 0.05, **p < 0.01, and ***p < 0.001 versus con group. (D) Immunofluorescence double staining for Iba‐1 (green) with CD16 (red) in SN on Days 1, 3, and 5 following MPTP injection. Quantification of the percentage of CD16+/Iba‐1+ cells. Immunofluorescence double staining for Iba‐1 (green) with Arg‐1 (red) in SN on Days 1, 3, and 5 following MPTP injection. Quantification of the percentage of Arg‐1+/Iba‐1+ cells. Scale bar = 20 µm. *p < 0.05, **p < 0.01, ***p < 0.001: MPTP group versus saline group.

Neuroinflammation is implicated throughout PD progression. In the 1‐methyl‐4‐phenyl‐1,2,3,6‐tetrahydropyridine (MPTP) mouse model, pro‐inflammatory cytokines are elevated in the substantia nigra (SN) and anti‐inflammatory treatments can mitigate dopamine neuron loss and behavioral deficits (W. W. Liu, Wei, et al. 2020; Lofrumento et al. 2011). We examined how pro‐inflammatory mediators change dynamically in the SN following MPTP administration. Our findings showed that levels of pro‐inflammatory cytokines (IL‐1β, IL‐6, and TNF‐α) progressively increased starting from Day 1, reaching their peak by Day 3 (Figure 1A). Given that activated microglia are categorized into proinflammatory M1 and anti‐inflammatory M2 subtypes (Yan et al. 2018), we characterized these populations in the SN following MPTP administration. RT‐PCR results revealed that M1 microglia markers (CD16, CD32, CD86, iNOS, CD11b) increased progressively from Day 1 and peaked between Days 3 and 5. In contrast, M2 microglia markers (Arg1, CD206, YM‐1, IL‐10, and TGF‐β) rose on Day 1 but began to decline after Day 3, returning to baseline levels by Day 14 (Figure 1B,C).

M1 and M2 genes are expressed in various CNS nerve cells. To determine microglial polarization following MPTP, we conducted double immunofluorescence staining with Iba‐1 in the SN, focusing on M1 and M2 marker proteins. The immunofluorescence findings aligned with the RT‐PCR data, showing elevated CD16 (an M1 marker) in microglia on Days 1, 3, and 5 following MPTP administration (Figure 1D). Conversely, the M2 marker Arg‐1 in Iba+ cells was notably increased compared to controls on Day 1, but began to decline by Day 3 (Figure 1D). These results indicate that M2 microglia were initially recruited to the SN after MPTP, though their presence was transient, with the M1 phenotype becoming predominant in the later stages.

### 3.2 Spermidine Pretreatment Reduced the Inflammatory Response and Modulated the Polarization of M1/M2 Microglia

**FIGURE 2:**
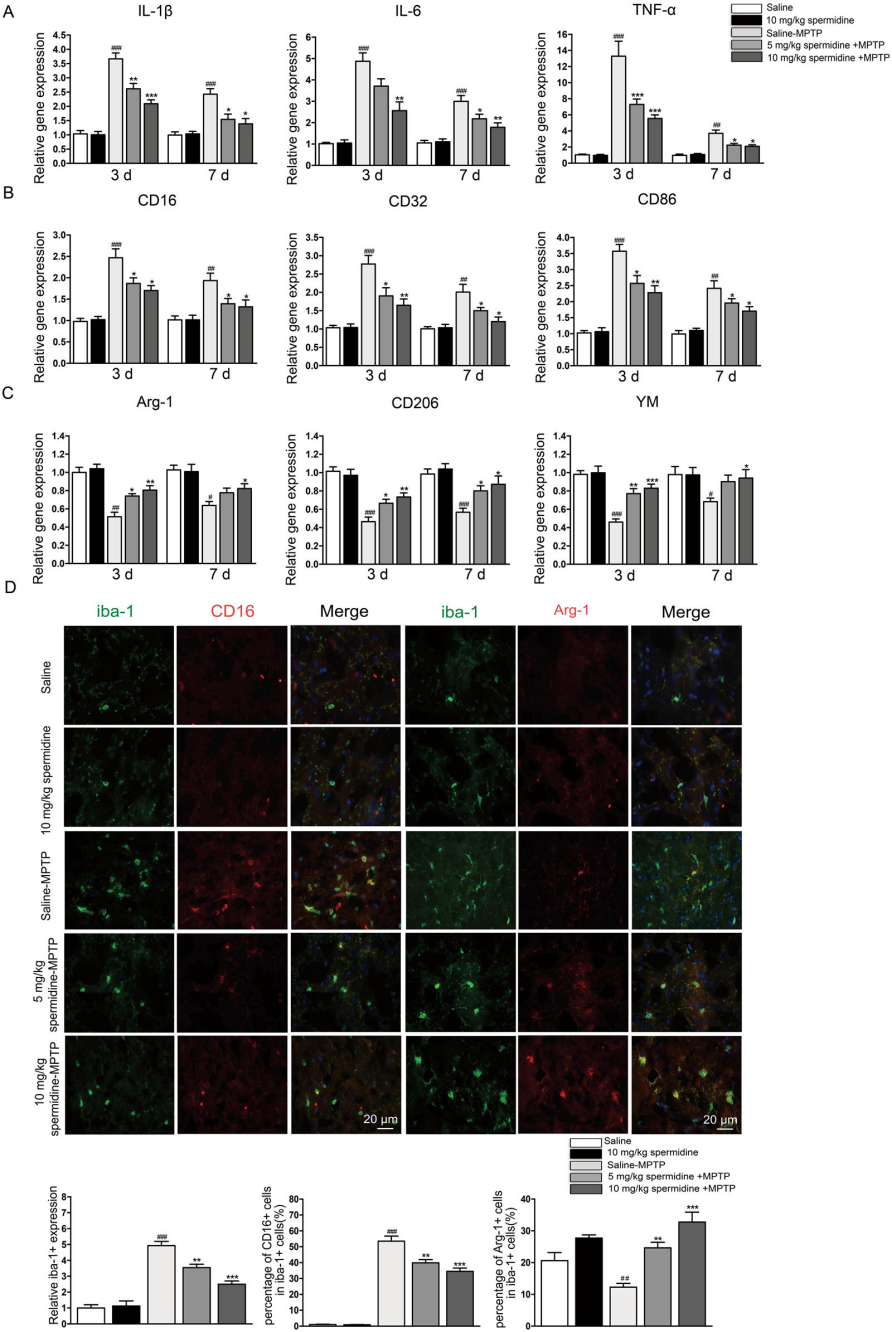
Spermidine pretreatment reduced the expression of M1 markers and increased expression of M2 markers in SN of PD mice. Change in expression of (A) IL‐1β, IL‐6, and TNF‐α; (B) CD16, CD32, and CD86 (M1 microglial markers); and (C) Arg‐1, CD206, and YM‐1 (M2 microglial markers) on Days 3 and 7 in SN of different group as determined by RT‐PCR. (D) Expression of Iba‐1 (green) with CD16 (red) or Arg‐1 (red) on Day 3 in SN following MPTP injection as determined by Immunofluorescence double staining. Quantification of microglial number in SN. Quantification of the percentage of CD16+/Iba‐1+ cells. Quantification of the percentage of Arg‐1+/Iba‐1+cells. Scale bar = 20 µm. ^##^ *p* < 0.01, ^###^ *p* < 0.001: saline‐MPTP group versus saline group; **p < 0.01, ***p < 0.001: spermidine‐MPTP group versus saline‐MPTP group.

Recent studies suggest spermidine has anti‐inflammatory effects (Jeong et al. 2018). However, its impact on microglia‐mediated neuroinflammation and M1/M2 polarization in PD remains unclear. To investigate, we conducted RT‐PCR on pro‐inflammatory cytokines in brain samples from PD mouse models sacrificed on Days 3 and 7 post‐MPTP injection. Spermidine treatment in MPTP mice reduced pro‐inflammatory cytokines (IL‐1β, IL‐6, and TNF‐α) compared to saline‐treated controls (Figure 2A). To assess if spermidine's anti‐inflammatory effects are linked to M1 and M2 microglia polarization, we analyzed M1 and M2 marker expression by RT‐PCR on Days 3 and 7 following MPTP treatment. In the untreated MPTP group, M1 markers (CD16, CD32, and CD86) were elevated, while M2 markers (Arg‐1, CD206, and YM‐1) were reduced (Figure 2B,C). In contrast, spermidine‐pretreated mice exhibited lower levels of M1 markers and increased levels of M2 markers on both Days 3 and 7 after MPTP administration (Figure 2B,C). The immunofluorescence results supported the RT‐PCR data, indicating that spermidine pretreatment decreased the expression of the M1 microglia marker CD16 while increasing the expression of the M2 microglia marker Arg‐1 on Day 3 following MPTP administration (Figure 2D).

**FIGURE 3:**
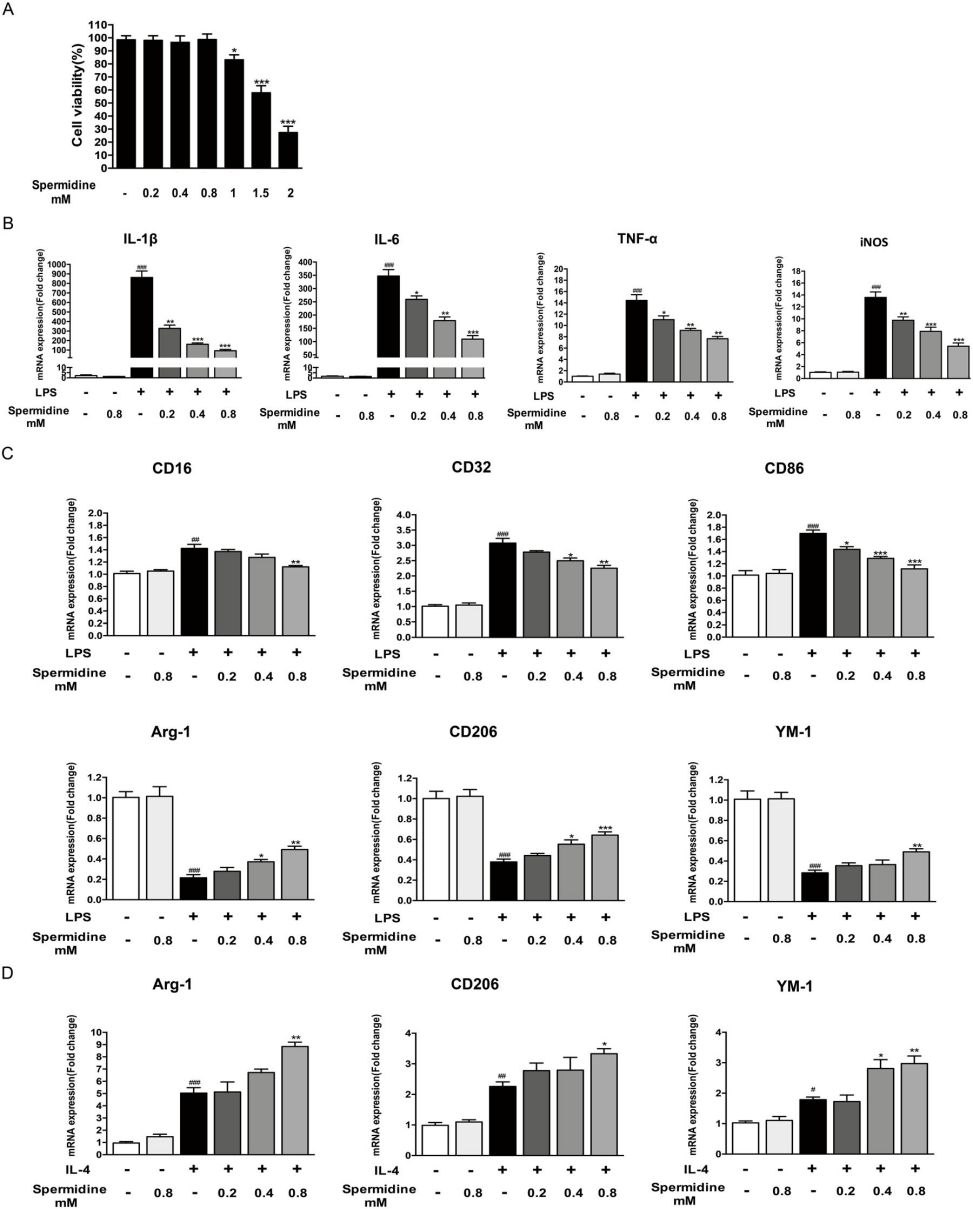
Spermidine reduced the mRNA expression of proinflammatory cytokines and M1 markers in LPS‐stimulated BV2 cells. (A) Effects of different concentrations of spermidine on the survival of BV2 microglia. *p < 0.05, ***p < 0.001, different concentration of spermidine group versus control group. (B) The expression of IL‐1β, IL‐6, TNF‐α, and iNOS were measured by RT‐PCR. (C) The mRNA expression of CD16, CD32, CD86 (M1 markers) and Arg‐1, CD206, and YM (M2 markers) in LPS‐induced BV2 cells as determined by RT‐PCR. ^#^ *p* < 0.05, ^##^ *p* < 0.01, ^###^ *p* < 0.001: LPS group versus vehicle group; *p < 0.05, **p < 0.01, ***p < 0.001: spermidine+LPS group versus LPS group. (D) The mRNA expression of Arg‐1, CD206, and YM (M2 markers) in IL‐4 induced BV2 cells as determined by RT‐PCR. ^#^ *p* < 0.05, ^##^ *p* < 0.01, ^###^ *p* < 0.001: IL‐4 group versus vehicle group; *p < 0.05, **p < 0.01: spermidine+IL‐4 group versus IL‐4 group.

In our vitro experiments, we investigated the impact of spermidine on inflammatory factor expression and the polarization of BV2 microglial cells. Initially, we evaluated the cytotoxicity of spermidine at various concentrations (0.2, 0.4, 0.8, 1, 1.5, and 2 mM). We found no cytotoxic effects up to 0.8 mM, so we selected concentrations below 0.8 mM for further experiments (Figure 3A). Our findings showed that spermidine at 0.2, 0.4, and 0.8 mM markedly decreased the mRNA levels of IL‐1β, IL‐6, TNF‐α, and iNOS in LPS‐stimulated BV2 cells, with the effect being dose‐dependent (Figure 3B). Notably, spermidine at 0.8 mM exhibited the most pronounced anti‐inflammatory effect.

**FIGURE 4:**
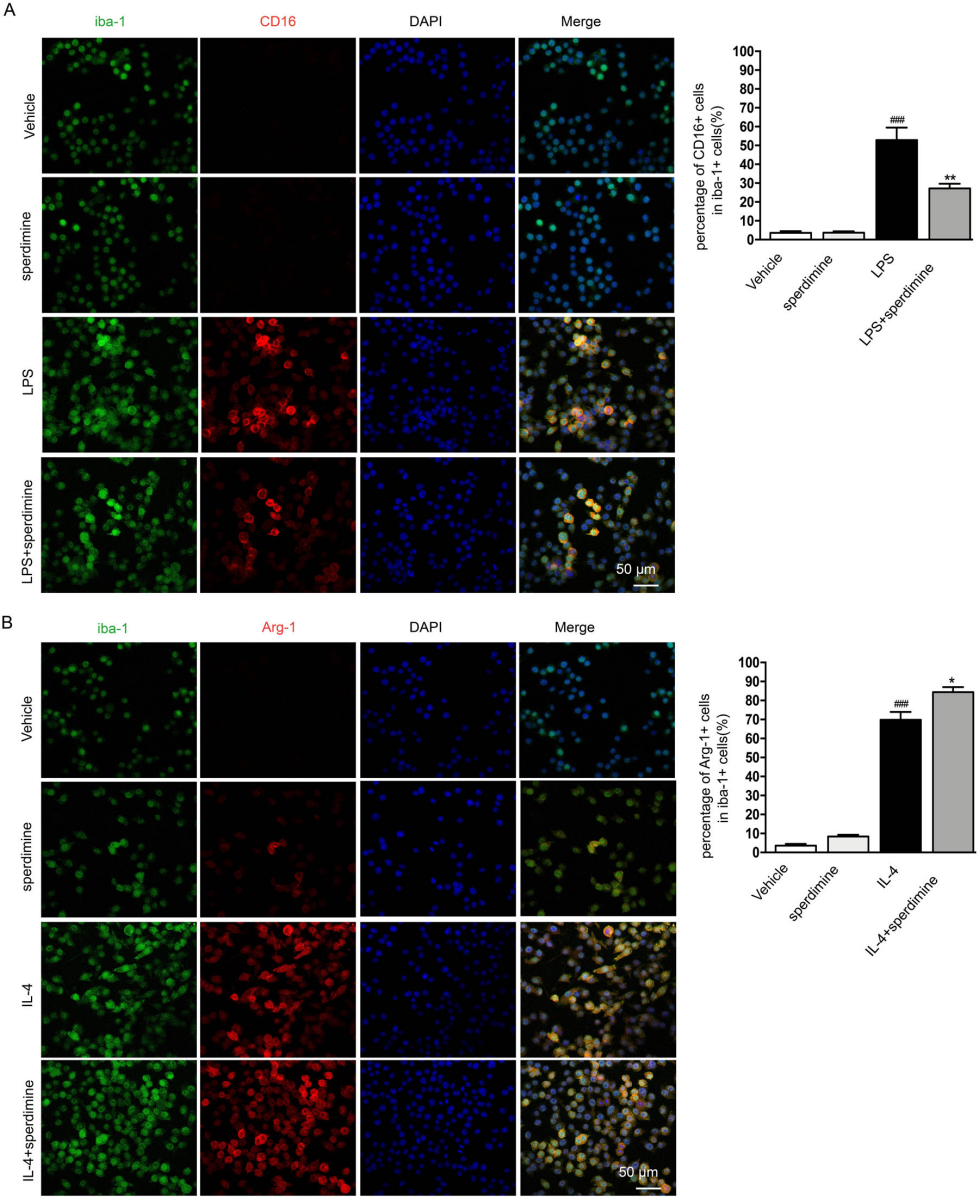
Spermidine reduced the expression of CD16 (M1 marker) and increased the expression of Arg‐1 (M2 marker) in vitro. (A) Expression of Iba‐1 (green) with CD16 (red) in LPS induced BV2 cells as determined by immunofluorescence double staining. Quantification of the percentage of CD16+/Iba‐1+ cells. ^###^ *p* < 0.001: LPS group versus vehicle group; *p < 0.05, **p < 0.01: spermidine+LPS group versus LPS group. (B) Expression of Iba‐1 (green) with Arg‐1 (red) in IL‐4 induced BV2 cells as determined by immunofluorescence double staining. Quantification of the percentage of Arg‐1+/Iba‐1+cells. ^###^ *p* < 0.001: IL‐4 group versus vehicle group; *p < 0.05, **p < 0.01: spermidine+IL‐4 group versus IL‐4 group. Scale bar = 50 µm.

In subsequent experiments, we assessed M1 and M2 marker expression in LPS‐ and IL‐4–activated BV2 cells using RT‐PCR and immunofluorescence. The results showed that 0.8 mM spermidine markedly decreased the expression of M1 markers (CD16, CD32, and CD86) and elevated the expression of M2 markers (Arg‐1, CD206, and YM‐1) in LPS‐stimulated BV2 cells (Figure 3C). This suggests spermidine promotes a shift toward the M2 phenotype. IL‐4 is recognized for encouraging the polarization of microglia toward the M2 phenotype, which is linked to anti‐inflammatory effects and tissue repair (Cherry et al. 2015). To further examine the potential effects of spermidine on M2 activity, BV2 cells were treated with IL‐4. This treatment led to an increase in the mRNA levels of Arg‐1, CD206, and YM‐1 (Figure 3D). Remarkably, pretreatment with 0.8 mM spermidine significantly boosted the expression of Arg‐1, CD206, and YM‐1 (Figure 3D). To further validate the findings from the previous experiments, the levels of the M1 microglia marker (CD16) and the M2 microglia marker (Arg‐1) were assessed using immunofluorescence. In line with the earlier results, pretreatment with 0.8 mM spermidine reduced the expression of the M1 marker CD16 in LPS‐stimulated microglia (Figure 4A) and enhanced the expression of the M2 marker Arg‐1 in IL‐4‐stimulated microglia (Figure 4B).

### 3.3 Spermidine Treatment Inhibited the p‐NF‐κB/p‐STAT1/p‐p38 MAPK Signaling Pathway and Activated the p‐STAT6 Signaling Pathway Both In Vivo and In Vitro

**FIGURE 5:**
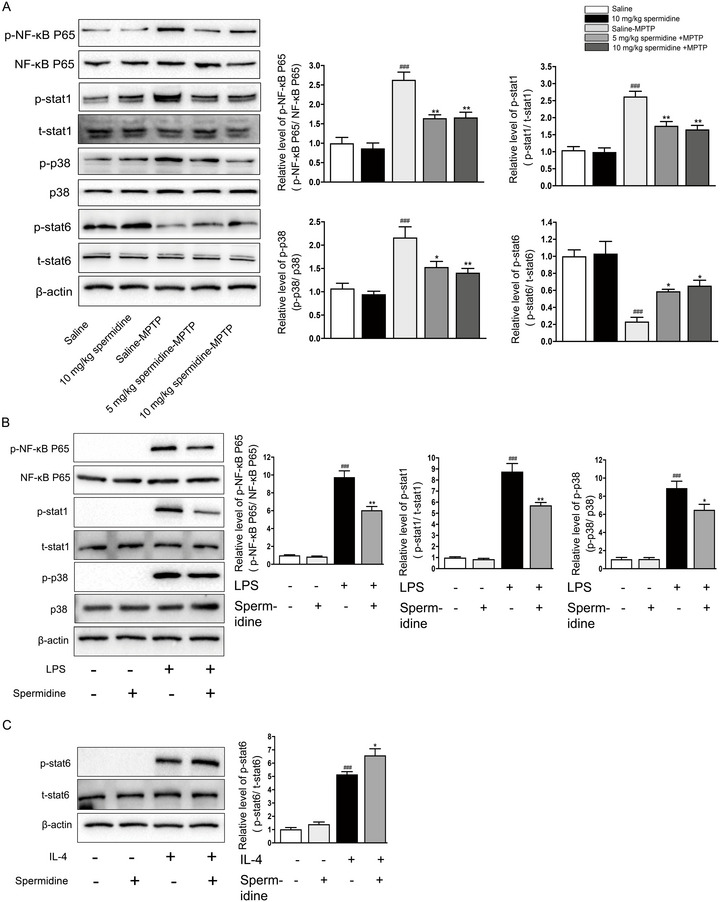
Spermidine inhibited the phosphorylation of NF‐κB p65, STAT1, and p38 MAPK, while it increased the phosphorylation of STAT6, both in vivo and in vitro. (A) Expression levels of P‐NF‐κB p65, NF‐κB p65, p‐STAT1, t‐STAT1, p‐p38 MAPK, p38 MAPK, p‐STAT6, and t‐STAT6 in SN were analyzed by western blotting on day 3 after last MPTP injection. Quantification of the densitometric value of the P‐NF‐κB p65, p‐STAT1, p‐p38 MAPK, p‐STAT6 protein bands is shown, normalized to NF‐κB p65, t‐STAT1, p38 MAPK, and t‐STAT6. ^###^ *p* < 0.001: saline‐MPTP group versus saline group; *p < 0.05, **p < 0.01: spermidine‐MPTP group versus saline‐MPTP group. (B) Expression levels of p‐NF‐κB p65, NF‐κB p65, p‐STAT1, and t‐STAT1, p‐p38 MAPK, p38 MAPK in different group were analyzed by western blotting. Quantification of the densitometric value of the P‐NF‐κB p65, p‐STAT1, and p‐p38 MAPK normalized to NF‐κB p65, t‐STAT1, and p38 MAPK. ^###^ *p* < 0.001: LPS group versus vehicle group; *p < 0.05, **p < 0.01: spermidine+LPS group versus LPS group. (C) Expression levels of p‐STAT6 and t‐STAT6 in different group were analyzed by western blotting. Quantification of the densitometric value of the p‐STAT6 normalized to t‐STAT6. ^###^ *p* < 0.001: IL‐4 group versus vehicle group; *p < 0.05: spermidine +IL‐4 group versus IL‐4 group.

Given that the NF‐κB, STAT1, p38 MAPK, and STAT6 pathways are crucial in regulating the M1/M2 balance in microglial cells (Bernardo and Fibbe 2013; B. Liu et al. 2021), we investigated the phosphorylation status of NF‐κB, STAT1, p38 MAPK, and STAT6 in the SN of MPTP‐treated mice. Western blot analysis revealed that MPTP injection led to the phosphorylation of NF‐κB p65, STAT1, and p38 MAPK, while it reduced the phosphorylation of STAT6 in the substantia nigra (SN) 3 days after the final MPTP administration (Figure 5A). In contrast, spermidine treatment inhibited the phosphorylation of NF‐κB p65, STAT1, and p38 MAPK enhanced the phosphorylation of STAT6 compared to the saline‐MPTP controls (Figure 5A).

Previous studies have demonstrated that phosphorylated NF‐κB p65, STAT1, and p38 MAPK play crucial roles in the polarization of M1 microglia in response to LPS stimulation (H. Li, Shen, et al. 2021; Zhong et al. 2020). To explore how spermidine reduces pro‐inflammatory factor expression and regulates microglial polarization, BV2 cells were pretreated with spermidine for 1 h before being exposed to 100 ng/mL LPS for 2 h. Western blot analysis was used to evaluate spermidine's impact on the activation of NF‐κB p65, STAT1, and p38 MAPK. The findings showed that LPS stimulation significantly increased the phosphorylation of NF‐κB p65, STAT1, and p38 MAPK (Figure 5B). However, pretreatment with spermidine markedly diminished the phosphorylation of NF‐κB p65, STAT1, and p38 MAPK that was induced by LPS (Figure 5B). Additionally, our study demonstrated that pretreatment with 0.8 mM spermidine enhanced the phosphorylation of STAT6 in microglia stimulated with IL‐4 (Figure 5C).

### 3.4 Spermidine Treatment Improved Behavioral Scores and Reduced Neuronal Cell Death in the MPTP‐Induced PD Model

**FIGURE 6:**
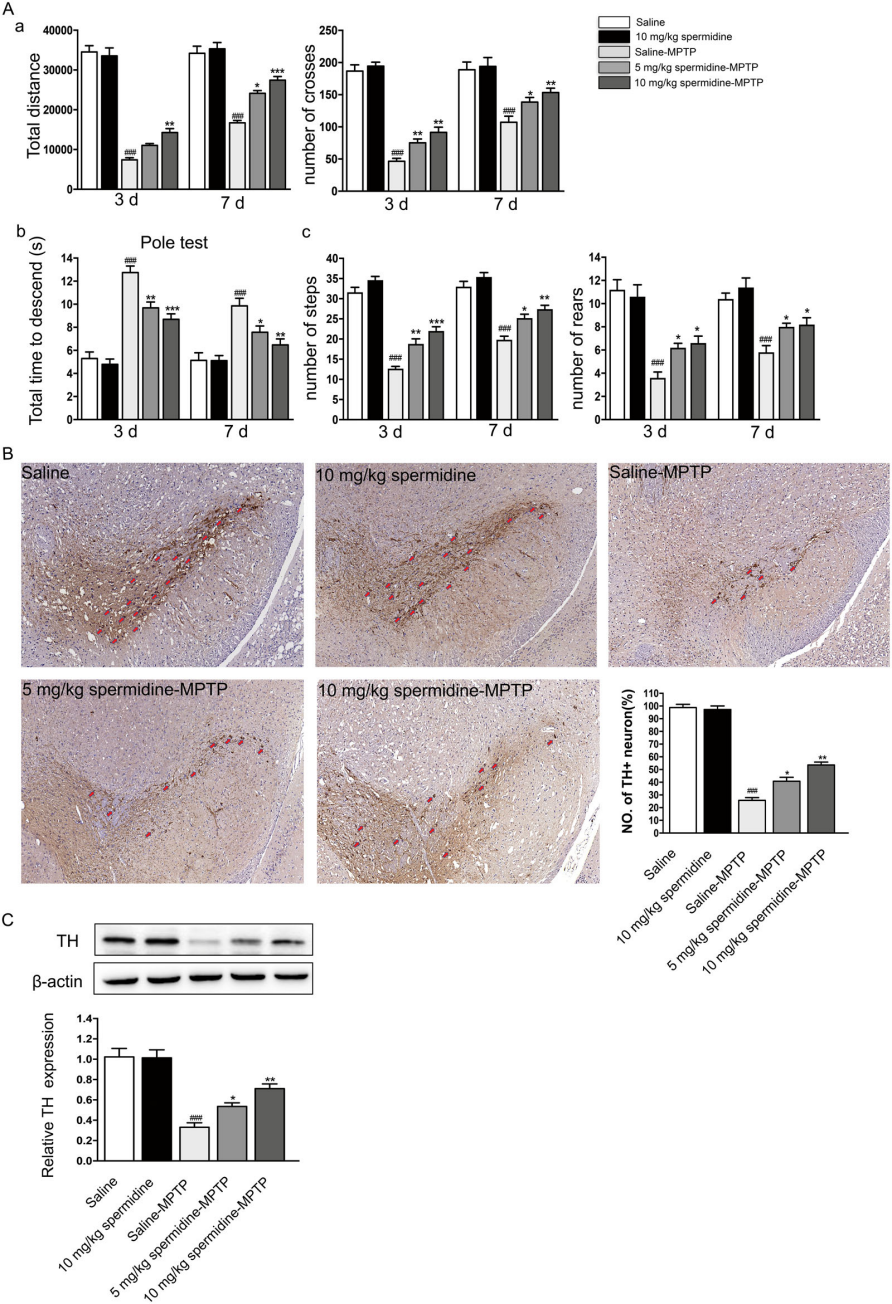
Spermidine ameliorated MPTP‐induced motor dysfunction and neuronal cell death in PD mice. (A) a. Total distance and number of squares crossed in the open field experiment were recorded in different experimental groups. b. In the pole test, the time each mouse reached the bottom of the pole was recorded and analyzed. c. In the staircase test, steps and rears were recorded in different experimental groups. (B) TH+ dopaminergic neurons in SN of PD mice were analyzed by immunohistochemistry. The red arrow points to the TH+ neuron. Number of the TH+ neuron (%) in the different experimental groups. (C) The protein expression of TH was analyzed by western blotting. ^###^ *p* < 0.001: saline‐MPTP group versus saline group; *p < 0.05, **p < 0.01, and ***p < 0.001: spermidine‐MPTP group versus saline‐MPTP group.

Next, we investigated whether spermidine treatment influences neurobehavioral damage and neuronal death in a PD mouse model induced by MPTP. We used the open field test, pole test, and staircase test to evaluate the mice's activity in a novel environment. The paths of the mice were mapped on Days 3 and 7, with recordings of total distance traveled and locomotion (square crossings) over a 5‐min period. Saline‐MPTP‐induced PD mice demonstrated significantly reduced locomotion on Days 3 and 7 compared to saline‐treated control mice, as evidenced by the number of squares crossed in the open field test (Figure 6Aa). PD mice pretreated with saline showed diminished movement in the open field (Figure 6Aa). In contrast, spermidine treatment significantly improved locomotor activity at doses of 5 and 10 mg/kg on both Days 3 and 7. In the pole test, spermidine‐MPTP‐treated mice descended more quickly than saline‐MPTP‐treated mice (Figure 6Ab). Staircase test is a behavioral test employed to assess motor function, which is often impaired in PD, as it requires the use of fine motor skills and good coordination (Ingram et al. 2021). Therefore, we used staircase test to assess the motor function of the mice. We found MPTP significantly decreased the number of steps and rears. Spermidine treatment increased the number of steps and rears at doses of 5 and 10 mg/kg on both Days 3 and 7 (Figure 6Ac).

Studies have demonstrated that TH‐positive (TH+) neurons are indicative of dopaminergic neurons in the brain. In our study, we assessed the proportion of TH+ neurons using immunohistochemistry. The saline‐treated control mice exhibited a high proportion of TH+ neurons, at 99%. In contrast, the proportion of TH+ neurons in MPTP‐treated mice was significantly reduced to 25% (Figure 6B). To evaluate the impact of spermidine on TH+ neuron survival, we compared the spermidine‐MPTP group with the saline‐MPTP group. Results indicated that 40%–53% of TH+ neurons survived in the spermidine‐MPTP group, compared to the saline‐MPTP group (Figure 6B). Additionally, Western blot analysis revealed decreased TH protein expression in the SN of saline‐MPTP‐induced mice, while TH expression was upregulated in the SN of spermidine‐MPTP‐treated mice compared to saline‐MPTP‐treated mice (Figure 6C).

### 3.5 Spermidine Reduced Inflammation‐Induced SH‐SY5Y Cell Death via the BDNF/TrkB‐PI3K/AKT Signaling Pathway

**FIGURE 7:**
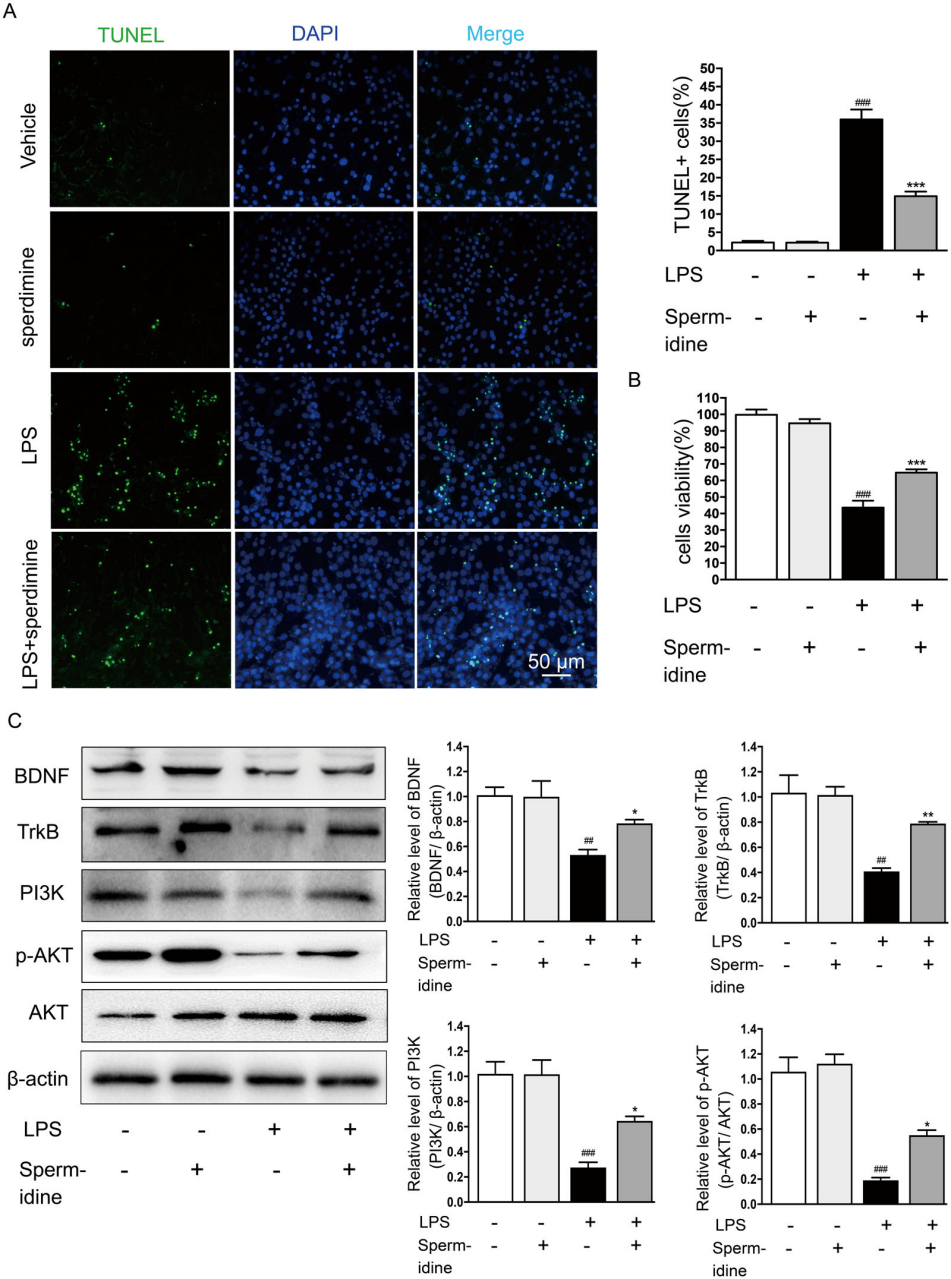
Spermidine indirectly protected SH‐SY5Y cells from death through the BDNF/TrkB‐PI3K/AKT signaling pathway. (A) TUNEL staining showed apoptosis of SH‐SY5Y cells pretreated with conditioned medium from different groups. Scale bar = 50 µm. (B) CCK‐8 assay kit was used to detect the effects of different groups of mediated conditioned medium from BV2 cells on the survival of SH‐SY5Y cells. (C) Expression levels of BDNF, TrkB, PI3K, and p‐AKT in different group were analyzed by western blotting. Quantification of the densitometric value of the BDNF, TrkB, and PI3K normalized to β‐actin. Quantification of the densitometric value of the p‐AKT normalized to AKT. ^##^ *p* < 0.01, ^###^ *p* < 0.001: LPS group versus vehicle group; *p < 0.05, **p < 0.01, and ***p < 0.001: spermidine +LPS group versus LPS group.

To explore spermidine's indirect effects on neuronal survival in vitro, we conducted experiments using SH‐SY5Y cells. We collected conditioned medium from various BV2 microglial cell groups and cultured SH‐SY5Y cells with this medium. Cell viability was assessed using TUNEL and CCK8 assays after 24 h. Conditioned medium from LPS‐stimulated BV2 cells significantly decreased SH‐SY5Y cell viability, while medium from BV2 cells pretreated with spermidine improved SH‐SY5Y cell survival (Figure 7A,B).

BDNF plays a crucial neuroprotective role by promoting neuronal survival, synaptogenesis, and brain repair through binding to its high‐affinity receptor, TrkB (Xiang et al. 2014). Furthermore, the activation of the BDNF‐TrkB receptor triggers the PI3K/AKT signaling pathway, which is widely expressed in the central nervous system, supporting neuronal survival, proliferation, and differentiation (C. Li, Sui, et al. 2021). However, it remains unclear whether spermidine exerts neuroprotective effects by activating the BDNF/TrkB‐PI3K/AKT pathway. Supernatant from LPS‐activated BV2 cells significantly decreased the protein expression of BDNF, TrkB, PI3K, and p‐AKT in SH‐SY5Y cells compared to the control group. These changes were significantly reversed by the conditioned medium pretreated with spermidine (Figure 7C).

## 4 Discussion

Polyamines like spermidine and spermine play crucial roles in regulating inflammation. They can modulate immune responses, influence cell proliferation, and impact inflammatory pathways, thus affecting overall inflammatory processes (T. T. Xu et al. 2020). During trauma, infection, and autoimmune diseases, polyamines such as spermidine and spermine increase in concentration at the site of inflammation, serving as part of an anti‐inflammatory response (Gobert et al. 2018). Spermidine pretreatment demonstrated a reduction in MPTP‐induced neurological function impairment and inhibited microglial activation. We found that spermidine effectively ameliorated neuroinflammation in the MPTP‐induced PD mouse model by modulating microglial function. Specifically, spermidine reduced pro‐inflammatory cytokine production in M1‐type microglia by inhibiting NF‐κB and STAT1 signaling pathways. Additionally, it enhanced anti‐inflammatory cytokine production in M2‐type microglia by activating STAT6 signaling. This dual modulation underscores spermidine's potential as a therapeutic agent in managing neuroinflammation associated with Parkinson's disease.

Microglia are key players in neuroinflammation. In both patients with Parkinson's disease and animal models of the disease, activated microglia are prominently found in the substantia nigra, a brain region critically affected by the condition (Kim and Joh 2006). Our research demonstrated that the levels of pro‐inflammatory cytokines increased progressively from Day 1, reaching their peak at Day 3. Similarly, the number of activated M1 microglia also rose steadily starting from the first day, peaking between Days 3 and 5 following MPTP administration. The count of anti‐inflammatory M2 microglia saw an increase on Day 1, but began to decrease 3 days after MPTP administration. Recent studies have indicated that spermidine, a polyamine involved in various cellular processes, exhibits notable anti‐inflammatory effects. Specifically, research has demonstrated that spermidine can modulate inflammatory responses by influencing several key mechanisms (Jeong et al. 2018; Yang et al. 2016). To explore the anti‐inflammatory effects of spermidine and its underlying mechanisms, we investigated its impact on microglial activation using an MPTP‐treated PD mouse model and BV‐2 cells. Our findings revealed that spermidine pretreatment reduced pro‐inflammatory cytokines on Days 3 and 7 post‐MPTP administration and significantly lowered the mRNA levels of these cytokines in LPS‐stimulated BV‐2 cells.

The role of activated M1 and M2 microglia in PD progression is intricate and multifaceted (L. Xu et al. 2016). M1 microglia, often described as pro‐inflammatory, contribute to neurodegeneration by secreting cytokines like TNF‐α, IL‐1β, and IL‐6, which exacerbate neuronal damage and promote inflammation. Conversely, M2 microglia are generally associated with anti‐inflammatory and neuroprotective functions, as they release cytokines such as IL‐10 and TGF‐β that help resolve inflammation and support tissue repair. However, the balance and timing of M1 and M2 activation can influence disease outcomes. Dysregulation or prolonged activation of either microglial subtype can lead to detrimental effects, complicating the understanding of their precise roles in PD. Thus, the dynamic interplay between these microglial states is crucial in determining the progression and severity of the disease. Therefore, future studies should explore the anti‐inflammatory mechanism of spermidine in relation to microglial polarization. Vivo et al. demonstrated that spermidine decreased the number of CD16‐positive cells while increasing the number of Arg‐1‐positive cells. This suggests that spermidine shifts microglial polarization away from the M1 phenotype and promotes a transition toward the M2 phenotype. Given the immunoregulatory role of spermidine, we examined its effects on microglia treated with IL‐4. Unexpectedly, spermidine pretreatment increased the expression of Arg‐1, CD206, and YM‐1 in IL‐4‐induced BV2 cells. This indicates that spermidine pretreatment facilitates the transition from the M1 to the M2 phenotype in microglia. Consequently, spermidine seems to mitigate inflammation by inhibiting the M1 phenotype and promoting the M2 phenotype.

Previous studies have shown that the inhibition of NF‐κB and STAT1 signaling pathways plays a crucial role in promoting the transition of microglia from the M1 to the M2 phenotype (Burke et al. 2013; L. Zhang et al. 2018). NF‐κB and STAT1 are key transcription factors involved in driving the inflammatory response associated with the M1 phenotype (Burke et al. 2013; L. Zhang et al. 2018). The attenuation of microglial activation by spermidine may be attributed to its inhibitory effects on the NF‐κB and STAT1 signaling pathways, which are crucial for the production of neuroinflammatory factors and the progression of PD. Our study observed a downregulation in the phosphorylation of NF‐κB and STAT1 in spermidine‐treated MPTP‐induced PD mice and LPS‐stimulated BV2 cells. This suggests that spermidine's modulation of these signaling pathways contributes to its anti‐inflammatory effects. Therefore, spermidine's inhibition of NF‐κB and STAT1 signaling may suppress M1 polarization in microglia during Parkinson's disease. Simultaneously, spermidine significantly upregulated M2‐type microglial markers, such as Arg1, CD206, and YM1, by activating the phosphorylation of the STAT6 pathway. Our study demonstrated that spermidine downregulated the NF‐κB/STAT1 pathway while promoting the STAT6 pathway in microglia. These effects may be linked to one or more of spermidine's functions. However, the precise mechanisms involved remain to be elucidated. Future research using knockout mice or specific signaling pathway inhibitors will be essential to clarify these underlying mechanisms.

Additionally, inducing the M2 phenotype in microglia can directly stimulate the synthesis of neurotrophins as well as the expression and function of their receptors, thereby promoting neuronal survival and preventing neuronal apoptosis (Song et al. 2013). We found that spermidine could indirectly inhibit the death of dopaminergic neurons in the substantia nigra of PD mice in vivo and reduce the death of SH‐SY5Y neurons in vitro. Our vitro study demonstrated that conditioned medium from LPS‐activated BV2 cells significantly reduced the viability of SH‐SY5Y cells and lowered the protein expression of BDNF, TrkB, PI3K, and p‐AKT. However, these effects were mitigated by conditioned medium from BV2 cells pretreated with spermidine. These findings suggest that spermidine may protect neurons by reversing the dysfunction of the BDNF/TrkB‐PI3K/AKT signaling pathway in damaged SH‐SY5Y cells.

Our research has some limitations. The MPTP‐induced PD model used is an acute neuroinflammatory model. Our study observed that pro‐inflammatory cytokines and M1 markers increased progressively from Day 1, peaked at Day 3, and returned to control levels by 14 days after MPTP injection. Consequently, we focused on assessing neuroinflammation at 3 and 7 days post‐MPTP injection with or without spermidine treatment. Data beyond 14 days post‐MPTP injection were not available. Indeed, Parkinson's disease is a chronic condition, and the symptoms induced in this PD model may not fully translate to the human disease. We aim to develop more advanced animal models in the future to better study neuroinflammation in Parkinson's disease. Furthermore, the anti‐neuroinflammatory effects of spermidine are not restricted to Parkinson's disease alone. For instance, Marina Jendrach's study demonstrated that spermidine pretreatment reduces neuroinflammation in an Alzheimer's disease mouse model (Freitag et al. 2022). This suggests that spermidine is not specific to neuroinflammation in Parkinson's disease. On the other hand, our study focused on the preventive effects of spermidine in the MPTP‐induced PD model and LPS‐stimulated BV2 cells. We also aim to explore spermidine's potential role in the treatment of neuroinflammation in Parkinson's disease in future research.

## 5 Conclusion

Previous studies have suggested that spermidine is one of the anti‐aging factors in humans (Madeo et al. 2019). Our study hypothesizes that the life‐extending effects of spermidine may be linked to its anti‐inflammatory activity. Our findings demonstrated that spermidine reduced M1 microglial polarization while promoting M2 polarization, potentially contributing to its anti‐aging effects. In summary, our study provides compelling evidence supporting the anti‐inflammatory role of spermidine. It suggests that the therapeutic effects of spermidine in Parkinson's disease are associated with the modulation of M1/M2 microglial polarization, indicating that spermidine could be a promising candidate for PD intervention.

## Author Contributions

**Jun Shu**: writing – original draft, methodology, data curation. **Yuqiong Jiao**: writing – original draft, data curation. **Wenshi Wei**: funding acquisition, supervision. **Aijuan Yan**: funding acquisition, writing – review and editing, project administration, supervision.

## Ethics Statement

The Ethics Committee of Huadong Hospital, affiliated with Fudan University, approved this study (2022JS‐087). All animal treatments and experimental procedures adhered strictly to the guidelines set by the Institutional Animal Care and Use Committee at Fudan University.

## Conflicts of Interest

The authors declare no conflicts of interest.

### Peer Review

The peer review history for this article is available at https://publons.com/publon/10.1002/brb3.70410.

## Data Availability

The data that support the findings of this study are available from the corresponding author upon reasonable request.
